# The effect of brushing with *Salvadora persica* (miswak) sticks on salivary *Streptococcus* mutans and plaque levels in children: a clinical trial

**DOI:** 10.1186/s12906-020-2847-3

**Published:** 2020-02-13

**Authors:** Heba J. Sabbagh, Khalil S. AlGhamdi, Hattan T. Mujalled, Sara M. Bagher

**Affiliations:** 1 Pediatric Dentistry Department, King Abdulaziz University, Jeddah, Saudi Arabia; 2 Bani Kabir Primary Health care Centre, Baljurshi Health Sector, Ministry of Health, Albaha, Saudi Arabia; 3 King Abdulaziz University, Jeddah, Saudi Arabia

**Keywords:** Miswak, Plaque score, Salvadora Persica, Salivary mutans *Streptococci*, And school children

## Abstract

**Background:**

The aim of the randomized double-blinded clinical trial was to evaluate the effect of tooth brushing with *Salvadora persica* (miswak) sticks on *Streptococcus* mutans count and the mean plaque score relative to brushing with fluoridated tooth paste (FTP).

**Methods:**

Our sample included 94 healthy, high caries-risk, 8 to 9-year-old students recruited from a government school, in Jeddah, Saudi Arabia between February and April 2016. Subjects were randomly grouped into test (provided with miswak sticks) and control groups (provided with FTP and soft brushes). Both groups were introduced to a preparatory period (PPP) of 3 weeks. Plaque score and saliva sampling were conducted prior to the PPP and in follow-up visits by a single, calibrated and blinded dentist.

**Results:**

Both groups showed a statistically significant decrease in the mean plaque score across the study (*P* = 0.007 and *P* = 0.001, respectively). In addition, subjects in the test group with abundant S. *sanguinis* increased from zero to six after 3 months.

**Conclusions:**

*Salvadora Persica* (miswak) and brushing with FTP significantly reduced plaque scores among school children. In addition, *Salvadora persi*ca was found to change the proportions of salivary bacteria in favor of species with less risk of inducing caries.

**Trial registration:**

ClinicalTrials.gov ID #: NCT04137393.

## Background

Dental caries is a microbial disease that originates from bacterial metabolic acid release and diffuses into enamel and dentine, dissolving the mineral [1]. Although the etiology of caries is multifactorial, mutans Streptococci (Streptococcus mutans, Streptococcus sobrinus) are reportedly the principal bacteria related to the disease [2]. Preventing and controlling dental caries can be accomplished by either oral hygiene measures affecting dental plaque (the main reservoir for oral bacteria) or a direct replacement of cariogenic with non-cariogenic colonies. However, such preventive measures may be limited to children of high socioeconomic status [3], while the dental disease burden of illness disproportionately affects children of lower socioeconomic status [4].

For thousands of years, plants have been used for dental hygiene and therapeutic practices [5]. The use of chewing sticks from *Salvadora persica* (miswak) plants is widespread in low socioeconomic areas in Asia, Africa, South America, and the Middle East, including Saudi Arabia [6]. Moreover, miswak is known as the “toothbrush tree”; studies reported that it contains fluoride, chloride, silica, and vitamin C, in addition to other healthy components [7].

Ezoddini-Ardakani et al., 2006 reported that *Salvadora persica* had a significant effect on preventing dental caries in second-year high school students in Iran [8]. Other studies reported a consistent association between the use of miswak and a decrease in oral bacteria [9–13]. However, a comprehensive assessment of the association between the plant and its prevention of dental caries among young children has yet to be conducted.

The present clinical trial therefore aimed to evaluate the effect of tooth brushing with *Salvadora persica* sticks on *Streptococcus* mutans count and the mean plaque score relative to brushing with fluoridated toothpaste (FTP) in a group of 8 to 9-year-old children at high caries risk.

## Methods

This randomized parallel double-blinded clinical trial was conducted at a local primary school in Jeddah, Saudi Arabia after the research proposal was approved by the research ethics committee of King Abdulaziz University Faculty of Dentistry (KAU), Jeddah, Saudi Arabia (066–16). It was registered on ClinicalTrials.gov with the ID number of “NCT04137393”.

### Subject selection

Our sample included 8 to 10-year-old male students recruited from Alshati Elementary Government School, in Jeddah, Saudi Arabia, between February and April 2016. The sample size was calculated using OpenEpi online sample size calculation (http://www.openepi.com/SampleSize/SSCohort.htm). The risk/prevalence difference was calculated according to Ezoddini-Ardakani et al., 2010, who reported that the difference in decayed, missing, and filled permanent teeth due to caries (DMFT) between individuals using miswak and those using a tooth brush was 55% [8]. This risk/prevalence difference gave us an effective sample size of 60 (30 test and 30 controls). However, to avoid sample regression in the follow-up visits, the sample size was increased to 94.

The criteria for inclusion were children at high-risk of developing caries with at least one cavity detected clinically by a trained dentist prior to participation in the study. All subjects with systemic disorders such as diabetes, hypertension, or sleeping disorders; subjects with regular use of medication; and children who received antibiotics during the month prior to the clinical examination were excluded from the study.

### Method

Prior to participating in the study, all children underwent a clinical examination (screening) administered by a trained dentist. Children diagnosed with at least one caries were considered to be at high risk based on the Caries Risk Assessment (CAT) [14] and thereby qualified for the study.

An educational lecture on oral hygiene and diet was provided to the children and class teachers by a single trained dentist. Arabic consent forms accompanied by a letter explaining the aim of the study were distributed to the parents of the qualified children. Parents who agreed to allow their children to participate were randomly allocated to either the test or control group classes using a coin toss. Participant enrollment and group assignments were carried out by the investigators with the help of external personnel assigned by the school administration. The distribution of children to cases and controls was concealed until the time of intervention with the external personnel.

The dentists visited the school after 1 week and distributed miswak sticks, uniform in length and width, supplied fresh from Makkah Miswak Central Market, Saudi Arabia, to the subjects in the test group. These miswaks were the roots of “Arak Siwak” plants that were originally plant in the south of Saudi Arabia. Subjects in the control group were supplied with FTP with a fluoride concentration of 1450 ppm and small, soft Oral B manual flat trim brushes.

In the test group, subjects were instructed to brush with the miswak using the rolling technique, advised to use it three times a day under a parent’s supervision, and shown how to keep it fresh by cutting off the edge of the miswak every day and storing it in the refrigerator at night. Subjects in the control group were also instructed in the rolling technique of brushing and advised to brush three times a day under a parent’s supervision.

To ensure that all included subjects received the same standard of care, both groups were introduced to a preparatory period (PPP) of 3 weeks. Saliva samples and plaque levels were obtained from the qualified subjects prior to the PPP (baseline). During the PPP, both groups performed daily supervised brushing. Morning sessions were monitored by two trained dentists from KAU in the first week and by the trained class teachers during the following 2 weeks; brushing was supervised by the class teachers. At the end of the PPP, plaque scores were obtained for the second time. Plaque scores were then assessed for the third time at the 3 months follow-up. Saliva samples were also obtained from the subjects at 1 month and 3 months follow-up.

The same trained dentists visited the school at least twice a week following the PPP for oral hygiene supervision. Extra tooth brushes and miswak sticks were provided in case any subject forget to bring his. Subjects were provided with a compliance chart and stickers. Each time the subject brushed at school, a sticker was placed by the supervising teacher. At home, the stickers were placed by the supervising parent. Only subjects who were available through the research period for school brushing were included in the study. Children who were absent for more than 5 days were excluded from the study. In addition, subjects who took antibiotics during the period of study were excluded from the study.

### Clinical examination

Examination for dental caries and plaque levels was conducted for the subjects during screening in optimal light using a mouth mirror and explorer by a single trained dentist. Diagnosis of dental caries was conducted based on the WHO criteria of 1987 and expressed as dmft for deciduous teeth and DMFT for permanent teeth (WHO 1978) [15]. Plaque levels were assessed using the Simplified Oral Hygiene Index by Green Vermillion (debris index) [16]. The examiner and laboratory were blinded to the subjects’ groups.

### Saliva samples

Saliva samples were collected from each participant for the assessment of salivary bacterial levels. Saliva sampling was performed prior to the PPP (baseline), at one and 3 months follow-up. The process was carried out between 7 and 11 am for a period of 15 min per subject. The subjects were instructed not to brush, eat, drink, or chew gum for 2 h prior to sampling.

### Microbiology assay

Literature was reviewed for microbes related to oral diseases, which include *Streptococcus* colonies [15, 17]. Accordingly, each saliva sample was examined using the quantitative colony count per colony forming unit (CFU/ml) *. The agar surface was wetted with saliva and then placed in a test vial, which was incubated at 37 °C for 48 h. The count of variance of all *Streptococcus* colonies was obtained. Findings of 10^5^ CFU or more of *Streptococcus mutans* indicated a high risk of developing caries, and findings of less than 10^5^ CFU, a low risk of developing caries. The frequency of subjects with high *Streptococcus* colonies was assessed. The laboratory technician was blinded to the subjects’ groups.

### Calibration

To reach good intra-examiner reliability, calibration of the examiner was conducted prior to the clinical examination. Ten randomly selected children were examined to assess their caries and plaque levels. Re-examination of the subjects was conducted after 15 days and the level of agreement between the two measurements was assessed using the Kappa method. We found 100% agreement with a kappa score of 1 (*p* < 0.001), indicating perfect agreement.

### Statistical analysis

The paired Samples T-test was used to compare between the mean plaque score of test and controls at different time periods (baseline, end of the PPP, and the 3 months follow-up). In addition, Chi-square test at a 0.05 level of significance, odds ratio (OR), and 95% confidence interval (CI) was used to compare between the frequencies of children with Streptococcus colonies of 10^5^ CFU or higher in the different study groups at different time periods (baseline, one, and 3 months follow-up).

## Results

**Fig. 1:**
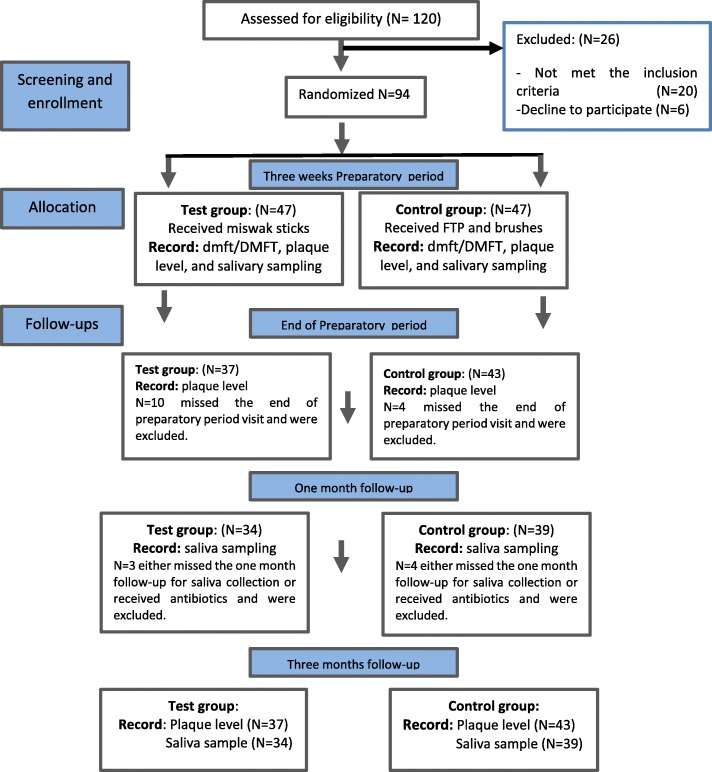
Flow chart for the study sample according to Consolidated Standards of Reporting Trials (CONSORT) guidelines

A total of 120 children were screened for the inclusion and exclusion criteria, resulting in 94 subjects enrolled in the study. Out of them, fourteen were absent for 5 days or more, and seven were excluded from the salivary bacterial analysis because they received antibiotics during the study period. This yielded 80 (37 test and 43 controls) subjects who were assessed for plaque levels and 73 (34 test and 39 controls) subjects who were assessed for salivary bacterial levels. Subject allocation and follow-up according to the Consolidated Standards of Reporting Trials (CONSORT) guidelines are illustrated in Fig. 1.

The dmft/DMFT mean score and standard deviation (± SD) for the test group was 7.4 (± 3.82), while that for the control group was 8.14 (± 3.94); no significant differences were found (*P* = 0.37).

The mean plaque score (±SD) (± SD) for the test group increased significantly after the PPP: from 1.31 (± 0.61) to 1.67 (± 0.62) (*P* = 0.04). On the other hand, the mean plaque score (± SD) for the control group increased from 1.53 (± 0.75) to 1.75 (± 0.68) over the same period; the difference was not statistically significant (*P* = 0.29). However, both miswak and control group subjects showed a statistically significant decrease in mean plaque scores (± SD) across the study (*P* = 0.007 and *P* = 0.00, respectively). In addition, there were no statistically significant differences in mean plaque scores (±SD) between the miswak and control groups prior to the PPP (*P* = 0.56), after the PPP (*P* = 0.44), or at the 3-month follow-up (*P* = 0.478) (Table 1).

**Table 1:** Comparison of mean plaque scores between baseline, after the preparatory period, and at the 3-month follow-up

Period of sample recruitment	Test group (***n*** = 37) ^**b**^	Control group (***n*** = 43) ^**b**^	***P*** value
Prior to the preparatory period (baseline)	1.3 (±0.61)	1.53 (±0.75)	0.56
After the preparatory period	1.67 (±0.62)	1.75 (±0.68)	0.44
***P*** value**	0.04*	**0.29**	
3-month follow-up	1.11 (±0.47)	1.29 (±0.47)	0.478
***P*** value***	0.007*	0.001*	

^**b**^less than the total number, children were absent (*N* = 14)

*Significant *P* value < 0.05

** Compared to the base line “prior to the preparatory period”

*** Compared to “after the preparatory period”

Regarding bacterial count, all subjects exhibited a high level of *Streptococci* colonies both prior to the PPP and after the PPP (more than or equal to 10^5^) (Table 2). In the test group, S. *mutans* had the highest frequency: 87.5% prior to the PPP. However, the frequency of S. *mutans* decreased to 73% and S. *sanguinis* increased to 13.5% after the PPP. At the 3-month follow-up, the S. *mutans* frequency increased to 82.4%. On the other hand, in the control group, the frequencies of S. *mutans*, S. *sanguinis*, and other bacteria were 76.7, 11.6, and 11.6%, respectively. After the PPP, the frequency of S. *mutans* increased to 83.7%, while that of S. *sanguinis* and other bacteria decreased to 7 and 9.3%, respectively. After the 3-month follow-up, both S. *mutans* and S. *sanguinis* frequencies increased to 89.7 and 10.3%, respectively (Table 2).

**Table 2:** Comparison between the bacterial count at baseline, after the preparatory period, and at the 3-month follow-up

Period	***S. mutans***	***S. sanguinis***	Other	Total	***P*** value
**Test group**					
Prior to the preparatory period (baseline) ^**b**^ **(*N*** **= 47)**	32 (86.5)	0	5 (13.5)	37 (100)	
1-month follow-up ^**b**^ **(*N*** **= 34)**	27 (73)	5 (13.5)	5 (13.5)	37 (100)	0.6
3-month follow-up ^**b**^ **(*N*** **= 34)**	28 (82.4)	6 (17.6)	0	34 (100)	0.88
**Control group**					
Prior to the preparatory period (baseline) ^**b**^ **(*N*** **= 47)**	33 (76.7)	5 (11.6)	5 (11.6)	43 (100)	
1-month follow-up ^**b**^ **(*N*** **= 39)**	36 (83.7)	3 (7)	4 (9.3)	43 (100)	0.078
3-month follow-up ^**b**^ **(*N*** **= 39)**	35 (89.7)	4 (10.3)	0	39 (100)	0.464

^**b**^ less than the total number, children were absent (*N* = 14) or received antibiotics during the study period (*N* = 7)

When comparing the test group to the control group in the three periods, we observed more subjects who experienced a decrease in the frequency of high *S*. *mutans* and an increase in S. *sanguinis* levels in the test group as compared to the control group (Table 3).

**Table 3:** Comparison between *Streptococcus-*colonies count at baseline, one, and 3-month follow-ups

Period		Test group n (%)	Control group n (%)	***P*** value OR (95% CI)
***Streptococcus*** **colonies**				
Prior to preparatory period (baseline) Test (*N* = 47) Control (*N* = 47)	*S. mutans*	32 (86.5)	33 (76.7)	a
	*S. sanguinis*	0	5 (11.6)	
	Others	5 (13.5)	5 (11.6)	*P* = 0.96 0.97 (0.25–3.67)
1-month follow-up Test (*N* = 34) Control (*N* = 39)	*S. mutans*	27 (73)	36 (83.7)	*P* = 0.3 0.45 (0.09–2.05)
	*S. sanguinis*	5 (13.5)	3 (7)	
	Others	5 (13.5)	4 (9.3)	*P* = 0.476 0.6 (0.15–2.45)
3-month follow-up*** Test (*N* = 34) Control (*N* = 39)	*S. mutans*	28 (82.4)	35 (89.7)	
	*S. sanguinis*	6 (17.6)	4 (10.3)	*P* = 0.36 0.53 (0.14–2.07)
	Others	0	0	a

a: not applicable because one of the input cells contains a value of 0

*** the total number (73) less than 80 because of children who were either absent or took antibiotics

## Discussion

Several studies have assessed miswak and its effect on oral health [9–13]. However, to overcome the complexity of studying oral bacteria [10], most studies were either conducted in vitro [11, 12] or assessed the effect of miswak on gingival health, which requires a relatively short time interval [13]. Thus, the literature features a dearth of high-quality studies on children, who require continuous assessment and supervision of oral-hygiene compliance. In addition, as miswak-use training is not included in the American Academy of Pediatric Dentistry (AAPD) anticipatory guidelines [18], children do not know how to efficiently use it for plaque removal. This study was therefore designed to overcome these limitations by recruiting children at an age when training for oral hygiene according to the AAPD guideline for oral hygiene (AAPD 2016) was possible and only supervision was needed (8 years old) [18]. In addition, a three-week PPP was arranged for oral hygiene training by two trained dentists; further, an ADA-based modified rolling method for brushing was also taught.

Our sample was selected from the Alshati elementary government school, where the children exhibited high dmft/DMFT, plaque scores, and mutans *Streptococci* levels, to assess the effect of miswak on children at high risk of developing caries. In addition, the children were all boys, who generally pay less attention to oral health care than girls do [19].

We found a significant preliminary increase in plaque score during the PPP in the test group. However, this increase was reversed significantly, and the children who used the miswak showed a similar ability to remove plaque as did those who used FTP after the 3 months of use. This indicates a lack of knowledge among children regarding the mechanical brushing method required by the miswak and the importance of education and training.

In addition, the number of children with an increase in S. *sanguinis* and a decrease in S. *mutans* rose after the use of the miswak, a finding that was not observed in the control group. As both S. *mutans* and S. *sanguinis* are related to the status of caries level in subjects, and the latter is associated with healthy tooth surfaces but not with caries [20, 21], our results suggest that miswak attenuated cariogenic bacterial colonies.

The findings of the present study therefore suggest that FTP could be replaced by miswak as an antiplaque and antibacterial agent. In addition, the results call for immediate action on the part of dentists to educate their patients on miswak use, especially in communities where miswak is a more convenient brushing method than FTP. This study also recommends the rolling technique for miswak use. Moreover, it introduces an oral health school program using miswak which helps to facilitate the performance of oral hygiene by children with no additional requirements other than obtaining the miswak stick itself. The most important strength of this clinical trial is its support of recommendations of consistent oral hygiene supervision and the 3-week PPP. Additional future studies with larger sample sizes and longer follow-up periods are needed, especially on medically compromised patients.

## Conclusion

*Salvadora persica* and bruising with FTP significantly reduced plaque scores among school children. In addition, *Salvadora persi*ca was found to change the proportions of salivary bacteria in favor of species with a lower risk of inducing caries.

## Data Availability

The datasets used and/or analyzed during the current study are available from the corresponding author upon reasonable request.

## Abbreviations

AAPD: American Academy of Pediatric Dentistry
ADA: American Dentistry Association
CAT: Caries-risk Assessment Tool
CFU: Colony Forming Unit
DMFT: Decayed, Missing and Filled Teeth
FTP: Flourided Tooth Paste
KAU: King Abdulaziz University
PPP: Preparatory Period
WHO: World Health Organization
